# Supply versus use designs of environmental extensions in input–output analysis: Conceptual and empirical implications for the case of energy

**DOI:** 10.1111/jiec.12975

**Published:** 2019-12-17

**Authors:** Hanspeter Wieland, Stefan Giljum, Nina Eisenmenger, Dominik Wiedenhofer, Martin Bruckner, Anke Schaffartzik, Anne Owen

**Affiliations:** ^1^ Institute for Ecological Economics Vienna University of Economics and Business Vienna Austria; ^2^ Institute for Social Ecology University of Natural Resources and Life Sciences Vienna Austria; ^3^ Institut de Ciència i Tecnologia Ambientals (ICTA) Universitat Autònoma de Barcelona (UAB) Bellaterra Spain; ^4^ School of Earth and Environment, University of Leeds Leeds UK

**Keywords:** energy consumption, energy efficiency, energy flow analysis, energy footprint, environmental input–output analysis, industrial ecology

## Abstract

Input–output analysis is one of the central methodological pillars of industrial ecology. However, the literature that discusses different structures of environmental extensions (EEs), that is, the scope of physical flows and their attribution to sectors in the monetary input–output table (MIOT), remains fragmented. This article investigates the conceptual and empirical implications of applying two different but frequently used designs of EEs, using the case of energy accounting, where one represents energy supply while the other energy use in the economy. We derive both extensions from an official energy supply–use dataset and apply them to the same single‐region input–output (SRIO) model of Austria, thereby isolating the effect that stems from the decision for the extension design. We also crosscheck the SRIO results with energy footprints from the global multi‐regional input–output (GMRIO) dataset EXIOBASE. Our results show that the ranking of footprints of final demand categories (e.g., household and export) is sensitive to the extension design and that product‐level results can vary by several orders of magnitude. The GMRIO‐based comparison further reveals that for a few countries the supply‐extension result can be twice the size of the use‐extension footprint (e.g., Australia and Norway). We propose a graph approach to provide a generalized framework to disclosing the design of EEs. We discuss the conceptual differences between the two extension designs by applying analogies to hybrid life‐cycle assessment and conclude that our findings are relevant for monitoring of energy efficiency and emission reduction targets and corporate footprint accounting.

## 1 INTRODUCTION

Industrial ecology is the study of the biophysical basis of human societies. Its interest lies in how socioeconomic systems extract, transform, use, and discard natural resources in order to produce, reproduce, and operate their biophysical structures (Pauliuk & Hertwich, 2015). Understanding the underlying mechanisms and drivers, for example, the role of household consumption or international trade, is a major concern for industrial ecologists. Input–output analysis (IOA) has proven to be a suitable framework in this regard (Duchin, 1992; Suh, 2010; Suh & Kagawa, 2006). To date, environmentally extended monetary IOA (Miller & Blair, 2009) is a common approach for the calculation of various attributional/footprint‐type indicators, such as consumption‐based or income‐based indicators (Malik, McBain, Wiedmann, Lenzen, & Murray, 2018; Marques, Rodrigues, Lenzen, & Domingos, 2012; Wiedmann & Lenzen, 2018).

Input–output (IO) model results can differ for many reasons, often rooted in the specifics of how they are constructed. IO tables can be divided into three overarching groups: Environmentally extended monetary IO tables (EE‐MIOT; Wiedmann & Lenzen, 2018), hybrid IO tables (HIOT) in mixed monetary and physical units (Merciai & Schmidt, 2018; Nakamura, Nakajima, Kondo, & Nagasaka, 2007), and physical IO tables (Hoekstra & van den Bergh, 2006; Kovanda, 2018). The different types of tables correspond to different layers of analysis (physical and/or monetary flows) implying different assumptions on prices and system boundaries (Schaffartzik, Wiedenhofer, & Eisenmenger, 2015; Weisz & Duchin, 2006). They can further have varying levels of geographical resolution or sector aggregation (Koning et al., 2015; Piñero, Heikkinen, Mäenpää, & Pongrácz, 2015) and, when constructed from supply–use tables (SUTs), use different technology assumptions, that is, constructs (Majeau‐Bettez, Wood, & Strømman, 2014). Through selection of different exogenous drivers, various IO models can be constructed from a given IO table, including supply‐driven (Ghosh, 1958; Marques et al., 2012) and demand‐driven IO models (Leontief, 1970), that treat capital formation either exogenously (Ivanova et al., 2016) or endogenously (Hertwich & Wood, 2018; Södersten, Wood, & Hertwich, 2018). Comparing results and elaborating the underlying conceptual differences and communalities therefore constitutes a constant point of discussion in the literature (Dietzenbacher, 2005; Eisenmenger et al., 2016; Hubacek & Giljum, 2003; Liang, Wang, Zhang, & Yang, 2017; Liang & Zhang, 2013; Suh, 2004).

The present work focuses on EE‐MIOTs and a rather neglected issue in recent IO model comparisons: the design of the environmental extension (EE) that accompanies the MIOT. An EE‐MIOT is an integrated dataset that combines two elements: a MIOT representing monetary inter‐industry flows and final demand, and an extension table in physical units depicting environmental flows that are associated with economic activities (UN, EU, FAO, OECD, & WB, 2017). Conceptually, extension tables are intended to describe flows required for economic activities that are not directly captured by the MIOT. The extension depicts flows “that cannot be fulfilled by the technosphere within a given time period” (Majeau‐Bettez, Wood, Hertwich, & Strømman, 2016, p. 69). This is analogous to factors of production, that is, inputs that are required for production but cannot themselves be produced in business establishments (Duchin, 1992). In environmental terms, this corresponds mostly to boundary flows entering (e.g., inputs from nature) or leaving (e.g., dilution of pollutants) the economy (UN et al., 2017). While efforts around the System of Environmental‐Economic Accounting (SEEA; UN et al., 2017) strive to provide a framework to consistently integrate environmental and economic information, in practice such data are usually not clearly matched. This obliges IO practitioners to make assumptions how the physical flows of the EE relate to the monetary flows of the MIOT (Schaffartzik et al., 2015). As this paper will illustrate for the case of EE‐MIOTs, the integration of monetary and physical accounts is a non‐trivial task (Lenzen, 2011) and the rationale behind it can vary significantly.

### 1.1 Extensions as a key source of variation in monetary IO models

EE‐MIOTs are the basis of global multi‐regional monetary IO (GMRIO) models, an IOA branch that is concerned with environmental footprints in general and especially environmental pressures embodied in international trade (Tukker, Giljum, & Wood, 2018; Wiedmann & Lenzen, 2018). Studies comparing GMRIOs revealed that differences in the EEs are the most important cause for differences in the footprints of nations, for example, the carbon footprint (Moran & Wood, 2014; Owen, Steen‐Olsen, Barrett, Wiedmann, & Lenzen, 2014; Owen, Wood, Barrett, & Evans, 2016; Tukker et al., 2018).1To the best knowledge of the authors, the only other environmental pressure for which similar comparisons are available is raw material extraction. Eisenmenger et al. (2016)) found that the differences in the material footprint of Austria, when calculated with various GMRIO models, could be partly explained by the differences in domestic material extraction, that is, the extension (compare with Tukker et al., 2018). Tukker et al. (2018)) traced differences in the extensions back to two key points: different source data and differences in definition and scope. The former points to the fact that different databases use different source data (e.g., energy or GHG emission statistics). The latter refers to the situation that extensions may use a different scope for the flows included. Usubiaga and Acosta‐Fernandez (2015) have shown that carbon footprint results can vary significantly when GHG extensions are constructed according to different accounting principles (territory versus residence), which is mainly due to different allocations of emissions from transport activities (road, water, and air). Furthermore, some studies neglect bunker fuels, which are used by international shipping and aviation companies, due to difficulties in data handling (Davis & Caldeira, 2010). But even when bunker fuels are included, the allocation issue is complex, requiring estimation methods, which impede a full understanding of the allocation to the MIOT (Peters, Davis, & Andrew, 2012). In general, the diversity in scope and definition in the extension design calls for a more transparent way of communicating which flows are included and how they are allocated to the MIOT, because this affects how results should be interpreted.

### 1.2 Different extension designs in energy IOA

The majority of studies that explicitly and empirically compared different extension designs can be found in the field of energy IOA2Miller and Blair (2009) present a wide array of different environmental IO models and thereby various options for integrating environmental data with monetary IO tables; for energy in particular (chapter 9) and environmental IO analysis in general (chapter 10). In chapter 10, the authors differentiate between models based on the generalized IO framework (see the model of the present study), fully integrated and limited economic‐ecologic models, and augmented Leontief models. However, for the sake of greater clarity, the authors present the different model types using stylized and simplified example tables and the comparative analysis of the model results is not their focus. (Owen et al., 2017). Two basic types of energy extensions are under discussion in the literature: an extension representing energy extraction and an extension representing energy use. The key difference between the two lies in the breakdown of the energy flow at different stages of the energy conversion chain and the attribution of flows, including transformation and transportation losses, to industries and/or final demands in the MIOT (Owen et al., 2017). The two designs thus reflect different measures of the distribution of energy inputs (resp. direct energy flows) to the socioeconomic system.

Costanza and Herendeen (1984) were the first to apply both extension designs in one study, however, the comparison was not the principal focus of their work. In recent years, both extension designs, energy extraction (Chen & Wu, 2017; Wu & Chen, 2017; Zhang, Qiao, Chen, & Chen, 2016) as well as energy use (Akizu‐Gardoki et al., 2018; Lenzen, 1998; Wachsmann, Wood, Lenzen, & Schaeffer, 2009; Zhang et al., 2015; Zhang, Zheng, & Fath, 2014), have been frequently applied. Motivated by renewed interest in consumption‐based energy accounting, Owen et al. (2017)) used a GMRIO model to undertake the first comparative analysis of energy footprints based on two different energy extensions. They find both designs useful and emphasize that they should be applied to different research questions.

### 1.3 Research gap, goal, and scope of the paper

Against this background, the aim of the present study is twofold: First, to reassess energy footprints resulting from two different EEs, an extension representing the energy supply to and another the energy use of the economy, in a single‐region IO (SRIO) framework. The present study therefore is a national counterpart to the GMRIO assessment conducted by Owen et al. (2017). We also take the assessment of Owen et al. (2017)) one step further by providing a detailed investigation of the implications for product‐level energy footprints. The main advantage of SRIO over GMRIO models is that they can be constructed from official statistics and are therefore fully compatible with the national accounts provided by national statistical authorities. Most GMRIO databases also use official national accounts as a starting point for further disaggregation and integration. However, national accounts are sometimes significantly modified in the course of the GMRIO compilation through balancing algorithms that are required to reconcile conflicting data (Edens et al., 2015; Hambÿe, Hertveldt, & Michel, 2018; Wilting, 2012).3Noteworthy in this regard is the single‐country national accounts consistent (SNAC) footprint approach where an SRIO table is combined with a GMRIO table to ensure that the footprints are derived from a dataset that is consistent with the official national accounts of a specific country (Edens et al., 2015). To focus our empirical analysis on the effects that stem from the decision for the extension design and to minimize the uncertainties involved, we derived both extensions from an official national energy supply–use account. Both extensions are then applied to the same SRIO model for one example country and different years (1999, 2007, and 2014). Austria was chosen as a case study because the authors are very familiar with the country and have a national IO model and detailed energy data at hand. Nevertheless, we crosschecked the SRIO findings with a GMRIO model and discuss energy footprints of regions calculated with EXIOBASE as well.

The second aim is to provide further clarifications of the empirical results by interpreting the outcomes of our comparison in light of the conceptual differences between the two extension designs. We apply analogies to hybrid life‐cycle assessments (LCAs) to discuss these conceptual implications. Inspired by the work of Pauliuk, Majeau‐Bettez, and Müller (2015), we use a graph approach to transparently describe different structures of extensions, that is, the scope of the physical flows and the attribution of these flows to industries and final consumers in the MIOT.

## 2 DIFFERENT EXTENSION DESIGNS FROM THE PERSPECTIVE OF ENERGY ACCOUNTING

### 2.1 Terminology and differences when using an SRIO versus a GMRIO framework

**Figure 1:**
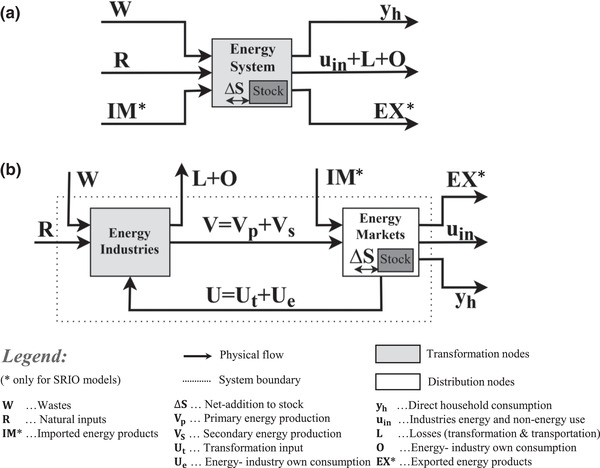
(a) Simplified visualization of the energy conversion chain where energy flows between industries and markets are depicted as a black box and only boundary flows that enter and leave the system are shown. (b) The energy conversion chain using a graph visualization of an ESUT. To facilitate a better overview, primary and secondary energy production processes are aggregated into one box. Adapted from Pauliuk, Majeau‐Bettez, and Müller (2015)

Below, we describe and visualize the two extension designs as they apply in an SRIO framework, as this is the primary basis of the empirical comparison in the present study. The main difference in the extension design of an SRIO and a GMRIO model lies in the presence of physical import and export flows of energy products, which are not explicit in the extension of a GMRIO, but need to be allocated in the EE of an SRIO model (compare with Owen et al., 2017 and see Figure 1). A supply‐extension for a GMRIO model thus only allocates energy extraction whereas an SRIO model also includes the allocation of physical trade flows. This is why we apply a slightly different and more generalized terminology than what has been used so far. What Owen et al. call the “energy‐extracted vector” is hereafter termed “supply‐extension.” As the present paper aims to use the nomenclature as set forth in the SEEA‐Energy framework, “extraction, which is used in material and energy flow accounting (Fischer‐Kowalski et al., 2011), is termed hereafter “natural inputs.”

### 2.2 The energy conversion chain

The key difference between the two EEs lies in the breakdown of the energy flow at different stages of the energy conversion chain and the attribution of flow industries and/or final demands in the MIOT (Owen et al., 2017). SEEA organizes physical flows in a series of tables and accounts, where physical SUTs play a key role. SUTs are balanced accounts of the total in‐ and outflows of industries and markets. Picturing the energy flows in an energy supply–use table (ESUT) enables a comprehensive description of the entire energy conversion chain, and hence all energy flows that are included in the two extensions (Heun, Owen, & Brockway, 2018). Further details regarding definitions and system boundaries in SEEA‐Energy and a detailed description of all energy flows depicted in Figure 1 are included in Supporting Information S2. Additional information on the graph approach and an extended visualization of the two designs is included in Supporting Information S2.

### 2.3 The two extension designs

A supply‐type extension allocates all energy flows that enter the ESUT (left side of Figure a), whereas the use‐type allocates all energy flows that leave the ESUT (right side of Figure a). When stock changes ($\Delta S\;=S_{\mathrm{add}}\;-S_{\mathrm{with}}$) are taken into account, the supply‐extension ($R+W+IM+S_{\mathrm{with}}$) and the use‐extension ($u_{\mathrm{in}}+y_{h}+S_{\mathrm{add}}+EX+L+O$) add up to the same totals.

A supply‐extension records energy flows at the entry point into the economy, representing the initial stage of the economy‐wide energy conversion chain. For example, *natural inputs* (*R* in Figure 1) such as crude oil, natural gas, and coal are allocated to mining industries, fuelwood to forestry, and hydropower to electricity. Energy from *wastes* (*W*) is allocated to the waste collection and treatment industries. The subsequent transformation and distribution steps, that is, the processing of *primary* into *secondary energy products* and the delivery of energy to final consumers (e.g., households), are fully incorporated/endogenized in the MIOT and thus follow monetary transactions. In contrast, a use‐extension represents flows at the final stage of the energy conversion chain. *Primary and secondary energy products* are allocated to the corresponding production (industries) and consumption entities (households) that consume energy. In Figure 1, this corresponds to energy industries’ own consumption ($O\;=U_{e}\;$), industries final energy use and non‐energy use (*u*_in_), transformation and transportation losses (*L*) as well as direct household consumption (*y*_h_). For example, bunker fuels are allocated to water transport and air transport, respectively; gasoline for private transportation to households, feedstocks; and process energy to the respective manufacturing industries. After this stage, energy is either released back to the environment when consumed (as losses of *residual* heat) or transformed into non‐energy products when used as a feedstock. The physical dimension of all transformation and distribution processes preceding the use‐phase is implicitly respected by the design of the use‐extension.

In the SRIO model of the present study, monetary transactions of imports are endogenized in the MIOT (see Section 3). This means they are treated as if they were produced within the (domestic) economy (see Section 3 and Section S3 in Supporting Information S2 for more details); hence they are included in the gross production vector of the monetary IO table. Consequently, imports (IM) of *primary and secondary energy products* are allocated to the respective production industries of the MIOT. For example, import of coal is allocated to mining and motor fuels to manufacturing of petroleum products. In the SRIO model of the present study, export flows of *energy products* (EX) are allocated directly to the final demand category of exports.

## 3 MATERIALS AND METHODS

### 3.1 The Energy Accounts

The Energy Accounts (EAs) of Austria that underlie our empirical calculations report the energy supply disaggregated by domestic and foreign origin and the energy use of 88 industries and households in a time series from 1999 to 2016 for 39 energy products (Statistics Austria, 2017). The use table shows the energy and non‐energy use of industries, losses, stock changes, exports, and the consumption of households. EAs are constructed according to the NACE 2008 industry classification, thus serving as a link between the System of National Accounts (EC, IMF, OECD, UN, & WB, 2009) and the Energy Balances of the International Energy Agency (IEA, OECD, & Eurostat, 2014).

**Figure 2:**
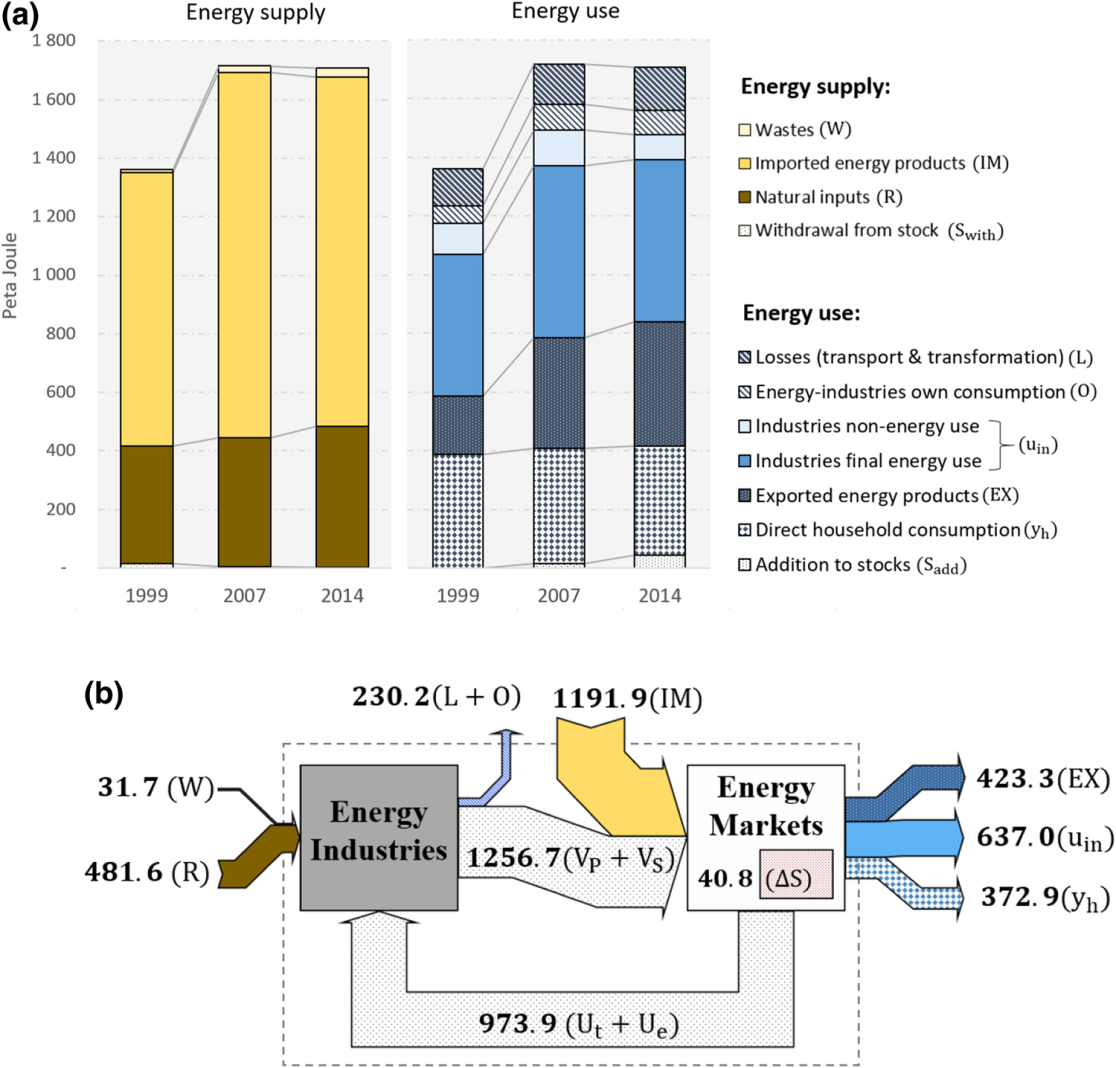
(a) Energy supply and use of Austria for 1999, 2007, and 2014. (b) Mapping the 2014 Energy Accounts according to the ESUT structure using Sankey flows. Underlying data can be found in Supporting Information S1. Unit: peta joule

**Figure 3:**
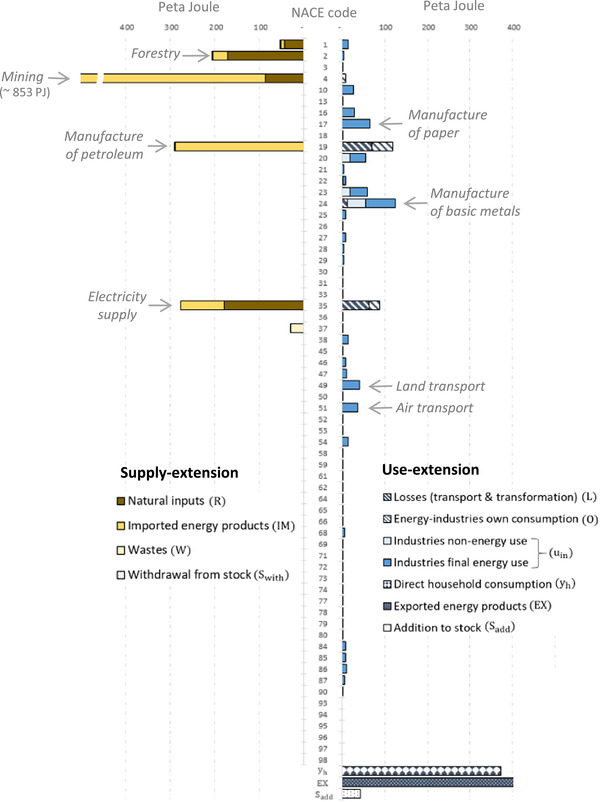
The two energy extensions for the year 2014 disaggregated by energy source and energy uses (including losses). Underlying data can be found in Supporting Information S1

To avoid double counting and to streamline the dataset with the classification of the SEEA‐Energy framework, industries’ intermediate energy use was disaggregated into non‐energy industries’ final energy consumption, energy industries’ own‐use (*O*), transformation output (*V*_S_), and transformation losses (*L*). A raw dataset that distinguishes these energy flows was provided by Statistics Austria and used for this purpose (Statistics Austria, 2018). Because official Energy Accounts do not provide an allocation of energy supply, energy loss, and non‐energy use to IO industries, adjustments were made, which are described in Section S6 in Supporting Information S2. Figures 2 and 3 provide an overview of the energy supply and use of Austria for the years 1999, 2007, and 2014.

### 3.2 The single‐region IO model

Monetary supply–use tables (MSUTs), which differentiate between 65 industries and 65 products, formed the basis of the SRIO model of Austria (Statistics Austria, 2014). Before constructing the IO model, the following two aggregation steps were required to match the MSUT and EA industry classifications. First, as EAs report *real estate activities* (code 68.A) and *imputed rents* (68.B) under one item, we aggregated the respective industries and products in the MSUTs. Second, we performed 15 many‐to‐one aggregations in order to align the more detailed EA classification (88 industries), with the MSUT classification (now 64 industries). The concordance table used for this purpose is included in Supporting Information S1.

Henceforth, capital and roman minor letters, respectively, denote matrices and vectors, while italic minor letters stand for scalars (single elements or running indices). The IO model construction followed the general description of the commodity‐by‐industry approach (Miller & Blair, 2009). An industry‐by‐commodity total requirement matrix was used:

$$L\;=\;D{\left(I-BD\right)}^{-1},$$

where *D*, the matrix of *commodity output proportions*, and *B*, the *commodity‐by‐industry coefficient* matrix, are derived from the basic MSUTs. A detailed description is included in Section S3 in Supporting Information S2. An element of matrix *L*, that is, $l_{ij}$ shows the value of output from industry *i* that is directly and indirectly required to produce one unit of commodity *j* for final demand. The IO model is then extended with direct energy coefficients

$$\;q^{k}=e^{k}\;\;{\hat{x}}^{-1},$$

representing energy flow *k* per unit of total industry production (*x*). The general format of the supply‐extended model therefore is:

$$\;F^{km}=\;\hat{q^{k}}\;L\;\hat{y^{m}}.$$

An element of matrix $F^{km}$, that is, $f_{ij}^{km}$ gives the amount of energy flow *k* that is embodied in intermediate input *i* of final product *j* purchased by final demand category *m*. For the implementation of the use design, the IO model needs to account for the energy used directly by final demand (households, exports, and stocks). This is achieved by adding an appropriate vector of direct energy flows (*c*) to the equation. The general format of the use‐extended model therefore is:

$$\;F^{km}=\;\hat{q^{k}}\;L\;\hat{y^{m}}+c.$$

Vector *c* follows the commodity classification of the IO model and its entries are non‐zero only for IO commodities representing energy products (Cruz, 2002).4This approach is discussed in detail in chapter 9 in Miller and Blair (2009).


IOA relies on the assumption of homogeneous prices, where products have the same price whether sold to industries, households, or to the export market. In fact, energy is not sold at the same price to all users, an issue that was already noted in early energy IO analysis (Bullard & Herendeen, 1975). Moreover, IOA assumes that products are homogeneous. In the case of the present SRIO model, this means that imported and domestically produced electricity are allocated along the same supply chains and there is no structural difference between supply chains that deliver household demand and export markets.

### 3.3 The global multi‐regional IO model EXIOBASE

In order to crosscheck the results from the SRIO model calculation with those generated by a GMRIO database, we apply EXIOBASE.5It should be borne in mind that differences between SRIO and GMRIO energy footprints for Austria (see Results section) can be explained partly by the different scope of the energy flows that are included. The SRIO footprints shown hereafter allocate only these energy flows to final demand that physically enter the domestic economy of Austria, whereas the GMRIO footprints also include energy that is used or consumed abroad, for example, the transformation losses embodied in the directly/physically imported energy commodity. Consequently, one can expect the household energy footprints to be larger when using a GMRIO database. National IO tables serve as the basic data source and starting point for further disaggregation, to represent and differentiate crucial sectors with environmentally sensitive activities (Wood et al., 2015). An industry–technology assumption is applied to transform the SUTs into symmetric input–output tables (Stadler et al., 2018). EXIOBASE version 3 distinguishes 200 products and 163 industries. In terms of regional detail, it has a clear focus on the EU. The EU‐28 and their 16 most important trading partners are explicitly modeled in EXIOBASE 3, representing about 95% of global GDP (Wood et al., 2015). The rest of the world (RoW) is aggregated into five separate “Rest of” regions. Overall, version 3 comprises 49 regions and countries. For the present study, the 2014 industry‐by‐industry IOT has been used. The EXIOBASE energy accounts are described in detail in supporting information 2 of Stadler et al. (2018).

## 4 RESULTS

This section presents and compares energy footprints when calculated with the supply‐extended and the use‐extended model. We start with results from the SRIO model of Austria and a description of the energy footprints of final products, followed by a comparison of the energy footprints of final demand categories. After that, we compare the energy footprint of regions when calculated with a supply‐extended and a use‐extended GMRIO model. In addition to Austria, here we also add 43 other countries and 5 “rest of the world” regions to the analysis. All results presented here only show energy footprints of 2014, the most recent year for which all data were available.

### 4.1 Energy footprints of final products

**Figure 4:**
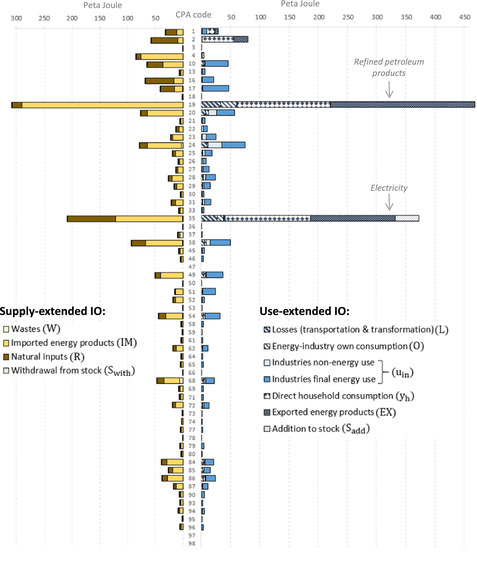
SRIO‐based energy footprints of 64 final products calculated using the supply‐extended (left) and the use‐extended model (right) for Austria 2014, broken down by the energy flows included in the respective extension. The footprints are aggregated across final demand categories. Underlying data can be found in Supporting Information S1

In Figure 4, we show the energy footprints of the 64 SRIO final products that we aggregated across all categories of final demand (households, non‐profit organizations, government, capital formation, and addition to stocks). Figure 4 shows that the supply‐extended and use‐extended model distributes energy to final products according to a similar pattern. Both models allocate most of the energy to refined petroleum products (CPA code 19), 307 PJ when using a supply‐extension and 466 PJ when applying a use‐extension, followed by electricity (code 35) with 208 and 373 PJ, respectively. These two final products thus have by far the largest energy footprint. The third largest product footprint in the supply‐extended model is construction (38) with 93 PJ and in the use‐extended model forestry (2) with 80 PJ. In general, we find that both IO models distribute energy in a comparable manner thus pointing toward the same final products, although with varying magnitudes. The product that deviates most strongly from this pattern is mining (code 4). Here, the use‐extended model allocates only 5 PJ (product rank number 35), while the supply‐extended model allocates 85 PJ (rank number 4).

Of the 64 product footprints, 44 are larger when using a supply‐extension, 17 when applying a use‐extension, and 3 footprints are identical because they are zero in both models (codes 47, 97, 98). Out of the 44 product footprints that are larger when using a supply‐extension, 30 refer to services, mainly shown in the lower part of Figure 4 (CPA codes 45 and higher).

**Figure 5:**
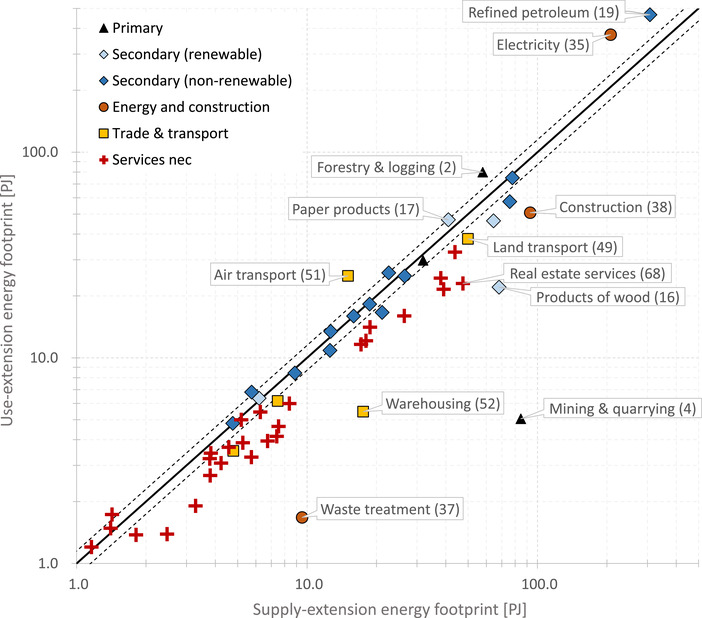
SRIO‐based energy footprints of final products from the supply‐extended model (*x*‐axis) and the use‐extended model (*y*‐axis), logarithmic scales, Austria 2014. The two dashed lines surrounding the equality line depict an indicative +/−15% deviation range. Product footprints that fall between these lines differ by not more than 15%. Labels show CPA codes. Unit: peta joule. Underlying data can be found in Supporting Information S1

The next figure illustrates the relative differences between product footprints. Figure 5 plots the energy footprints of the supply‐extension on the *x*‐axis and the footprints of the use‐extension on the *y*‐axis. A list assigning products to product groups is included in Supporting Information S1. Of the 64 product footprints, 15 are within the indicative 15% range of deviations (not counting the three product footprints that are zero), of which 10 belong to manufacturing (secondary products), 4 to services, and 1 to the primary sector (products of agriculture). The figure shows that energy footprints of services have the tendency to be larger when applying a supply‐extension (32 are larger when using a supply‐extension and 7 are smaller). The footprints with the largest relative deviation are mining and quarrying (code 4), where the supply‐extension footprint (87 PJ) exceeds the use‐extension footprint (5 PJ) by a factor of 16.7; waste treatment (37) with a factor of 5.7 (9 PJ and 2 PJ); warehousing services (52) with a factor of 3.2 (18 and 5 PJ); and products of wood (16) with a factor of 3.1. The large absolute deviations in the footprints of refined petroleum products (19) and electricity (35) become smaller when viewed in relative terms; the use‐extension exceeds the supply‐extension footprint by a factor of 1.5 and 1.8, respectively, for these products. Figure 5 indicates that, when applying a use‐extension, the larger amount of energy allocated to refined petroleum and electricity has its counterpart in the relatively smaller amount allocated to services, manufacturing, and other products.

### 4.2 Energy footprints by final demand categories

**Figure 6:**
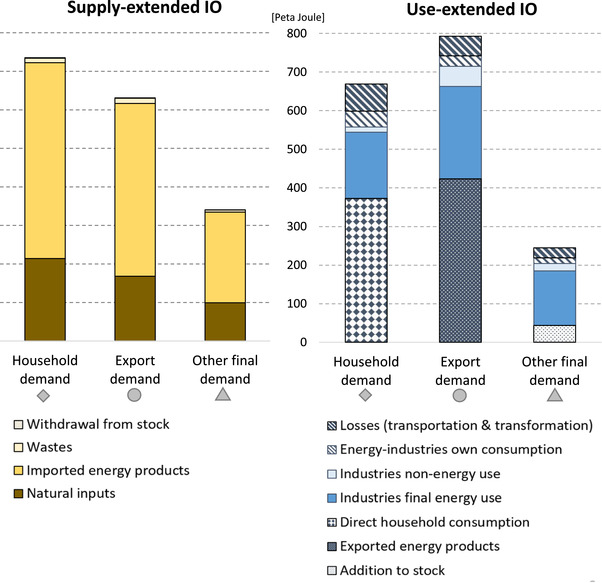
SRIO‐based energy footprints by final demand categories and by energy flows, 2014; supply‐extended model (left) and use‐extended model (right). Underlying data can be found in Supporting Information S1

We now investigate the energy footprints disaggregated by the different categories of final demand. The strongest convergence between supply‐extended and use‐extended model results, viewed in both relative and absolute terms, is found for the energy footprints of households. The supply‐extended model allocates 735.4 PJ to the final demand of households and the use‐extended IO model 669.1 PJ, resulting in a difference of 66.2 PJ. Taking a hypothetical mean of the two model results as a benchmark ( $\bar{x}=\left(735.4+669.1\right)/2\;=\;702.3)$, then household footprints differ by around 9.4% of that mean (see Figure 6). In comparison with the absolute and relative differences of exports (161.8 PJ or 22.7%) and the other final demand categories (98 PJ or 34%), this is the smallest value. The variation in the energy footprint of other final demand can be attributed foremost to differences in the footprint of gross fixed capital formation, with 190.1 PJ when using the supply‐design and 110.3 PJ when applying a use design, and government consumption, with 121.3 and 74.7 PJ, respectively. The strongest divergence in absolute terms is found for the energy footprint of exports; here the supply‐extended model allocates 631.1 PJ and the use‐extended model 792.9 PJ. The large absolute variation in export footprints, as can be seen in Figure 5, is to the largest part driven by the variations in the final product footprints of petroleum products and electricity. Section S5 in Supporting Information S2 provides a more detailed analysis of the energy footprints of final demand broken down by final products.

The relatively strong divergence in the export footprints leads to different rankings of the footprints of the final demand categories. When applying a supply‐extensions, most energy is allocated to households (735.4 PJ; 43.1% of energy supply), followed by *exports* (630 PJ; 37%) and *other final demand* (340.5 PJ; 19.9%). When applying a use‐extension this changes and most energy is allocated to exports (792.9 PJ; 46.5%), followed by households (669.1 PJ; 39.2%) and *other final demand* (244.8 PJ; 14.3%). The ranking of the footprints of the final demand components is thus sensitive to the extension design. Section S4 in Supporting Information S2 includes a comparison of the final demand footprints and the associated variations for the years 1999, 2007, and 2014, revealing a relatively stable pattern, that is, ranking over time.

### 4.3 Energy footprint of regions

**Figure 7:**
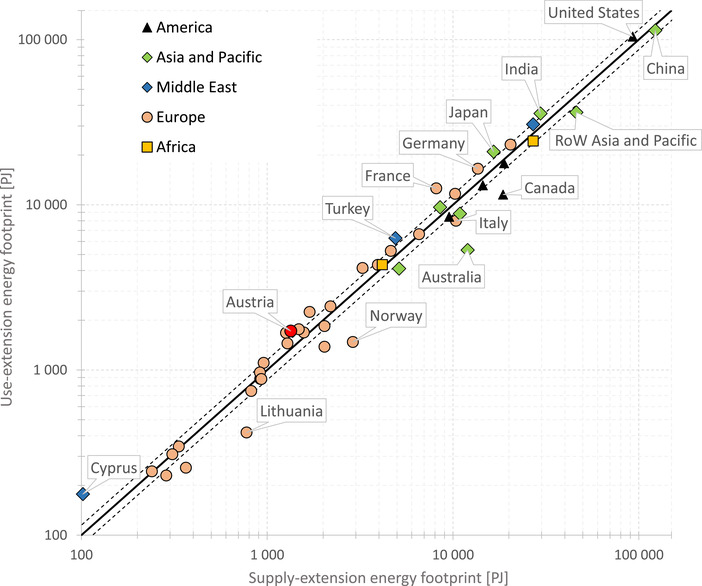
Supply‐extended and use‐extended energy footprints of nations, 2014, EXIOBASE. The two dashed lines surrounding the equality line depict the +/−15% range of deviations between the two designs of extensions. Underlying data can be found in Supporting Information S1

In order to validate the SRIO results against those generated with a GMRIO model, we calculated regional energy footprints with EXIOBASE. The aggregated energy footprint on the regional level shows direct and indirect energy use of the countries’ total final demand. Besides households, this also includes capital formation, government consumption, and other final demand categories, but excludes demand for exports. Summation of all supply‐extended and all use‐extended regional energy footprints must add up to the same total, that is, global inputs from nature (573,509 PJ in 2014). For total final demand of Austria, the supply‐extended footprint is approx. 1,339 PJ and the use‐extended footprint is 1,722 PJ. Taking the hypothetical mean of the two results, then the two energy footprints differ by approx. 25%. As can be seen in Figure 7, our analysis revealed that 24 of the 49 region footprints are outside the 15% threshold.

The largest absolute variations between supply‐extended and use‐extended energy footprints are observed for the United States (approx. 11,486 PJ), RoW Asia and Pacific (9,880 PJ) and China (approx. 9,028 EJ). The two largest relative variations are found for Australia, where the supply‐extended footprint (11,981 PJ) is more than twice (factor 1.25, i.e., approx. 125% larger) the use‐extended footprint (5,319 PJ), and Norway, where the supply‐extended footprint amounts to 2,888 PJ and the use‐extended footprint to 1,478 PJ hence the supply‐extended footprint exceeds its counterpart by a factor of 0.95. All EXIOBASE results are included in Supporting Information S1.

## 5 DISCUSSION

### 5.1 Empirical implications

Our empirical comparison revealed that SRIO‐based energy footprints are sensitive to the extension design, leading to different rankings of the footprints of the final demand categories. Questions may arise whether the supply‐extension and use‐extension footprints would converge more strongly when compared in a GMRIO framework. We therefore crosschecked our observations with energy footprints of regions calculated with EXIOBASE, with the results confirming the insights from the SRIO model comparison. We therefore conclude that energy footprints are sensitive to the extension design, not just in an SRIO but also in a GMRIO framework.

The comparison of SRIO results at the product level has shown, even more pronounced, that footprints can vary by several orders of magnitude. Only 15 of the 64 product footprints show deviations of less than 15%, which concerns particularly manufactured products (Figure 5). Much greater percentage deviations appear for a few key products (e.g., electricity, refined petroleum, and mining) that have pivotal importance for the energy supply and use of the economy. As IO‐based analysis are increasingly used to inform policy and demand‐side approaches to climate change mitigation, these differences are highly relevant in terms of prioritization. These variations are relevant especially for hot‐spot analyses in the context of corporate responsibility efforts (Kjaer, Høst‐Madsen, Schmidt, & McAloone, 2015; Martinez, Delgado, Martinez Marin, & Alvarez, 2018a, 2018b; Martinez, Marchamalo, & Alvarez, 2018), where EE‐MIOTs, also in hybrid LCA‐IOA frameworks (Suh et al., 2004; Yu & Wiedmann, 2018), are increasingly applied for calculating footprint accounts of organizations, companies, and products.

Moreover, our comparison showed that energy footprints of service sectors tend to be larger when applying a supply‐extension (Figure 5). We found that 32 services are larger when using a supply‐extension and 7 are smaller. We therefore conclude that energy footprints of services, when calculated with EE‐MIOTs, are structurally biased by the selection of the extension design. This finding gains in importance as service sectors are repeatedly discussed as a vehicle for green growth, the sharing economy, and recently for a circular low‐carbon economy (Kjaer, Pigosso, Niero, Bech, & McAloone, 2018; Lifset, 2000). This highlights that the analysis of the environmental implications of services should take special care to critically reflect on the respective extensions design used.

### 5.2 Conceptual implications

The system boundary of supply‐extended and use‐extended EE‐MIOTs are in principle the same, following standards in the system of national accounts. While the MIOT represents all flows of goods and services that have an economic, that is, market value, both energy extensions record the same energy flow through the system. However, depending on one's perspective, monetary supply chains and energy conversion chains intersect at different points, making an explicit assumption on allocation necessary.

The supply‐extended model implies a logic that is conceptually most strongly related to the partition allocation of environmental burden by economic value in traditional LCA (Reap, Roman, Duncan, & Bras, 2008; Schaffartzik et al., 2015). The energy supply assigned to the energy industries is distributed to downstream industries according to their monetary payments, given IOAs homogeneity assumptions on products and prices (recall the IO model description in chapter 3).

The use‐extended model resembles a model structure that is similar to tiered hybrid techniques in LCA‐IOA. In a tiered hybrid model, the system under study is partitioned into supplementary modules. The direct and some important lower‐order upstream energy requirements are examined in a separated (process analysis) module while remaining higher‐order requirements, for example, energy used in the extraction and processing, are covered by an input–output table (Suh et al., 2004). The use design facilitates a similar conceptual division. The first‐order energy use is captured by allocating direct energy use, for example, of fuels, to final consumption of households, exports, government, and investment already in the EE independently from monetary transactions or any assumptions on prices, using energy statistics.6In general, the IEA collects data for the energy balances based on standardized questionnaires (IEA, 2019a) that are submitted by national statistical institutions (NSI). The surveys include guidance on how the NSIs can or should collect the data just for a few important processes, for example, for combined heat and power plants (CHP). The methods for the data collection can therefore differ between countries. Data on monetary transactions are not a requirement for the compilation of energy consumption but could be used when only partial information is available. In the case of Austria, for example (Statistics Austria, 2016), an important data source for estimating the energy consumption of industries and households is sample surveys that are conducted every 2 years that are then combined with reports from market regulators such as “E‐Control” and other data sources. To the best knowledge of the authors, the claim that energy statistics are compiled independently from monetary transactions is true for the case of Austria but would need to be checked for other countries. The MIOT is then used to estimate and allocate the remaining higher‐order energy requirements, where in the environmental extension all the second‐order energy use is allocated to the respective energy‐using industries. Transformation losses7Note that though the present use‐extended IO model allocates transformation losses to the IO industries producing the energy. It is equally feasible to allocate the transformation losses directly to the energy users (households, exports, non‐energy industries), for example, assuming a constant ratio between final energy consumption and associated losses across users. This would result in removing the second‐order energy requirements (energy for processing) from the MIOT. This approach has been adopted by Owen et al. (2017). from producing energy commodities and energy industries’ own consumption then represent a third‐order energy use and are allocated through the MIOT along the respective supply chains to final demand. The use‐extended model setup therefore utilizes an environmental extension with two supplementary modules, where direct energy use of final demand is allocated through a data‐driven, often physical module from energy statistics, while the monetary (IO) module is used to account for all higher order, that is, indirect, energy requirements, by that means expanding the system boundary of the physical module.

### 5.3 Interpreting disagreement from a conceptual point of view

Following our conceptual considerations, we suggest to conceive energy footprints when calculated with a supply‐extension and a use‐extension as two different indicators. The observed divergences should not be solely interpreted as “errors” that are grounded in the homogeneity assumptions of IOA. Instead, the two indicators are derived from EE‐MIOTs with different system structures and hence are correct in their own regard. The supply‐extended footprint gives more weight to the monetary dimension and thus strongly reflects the economic drivers that push energy supply. Energy industries generate revenues by producing and selling energy. In this regard, the monetary expenditures of final consumers are viewed as an incentive for supplying energy. The use design puts more weight to the physical dimension of production and consumption, by partitioning the energy supply into its different economic uses and sectoral production “recipes,” making sector‐level technology mixes visible and clarifying the direct energy intensity of each sector and activity. Although the result of both EE‐MIOTs are described using the same terminology, they must be interpreted as providing different types of information.

### 5.4 Interpreting disagreement with regard to homogeneity assumptions

The SRIO model of Austria differentiates between 64 industries and products, where all monetary flows related to extraction of energy and non‐energy products, that is, metallic and non‐metallic minerals are aggregated into a single sector “mining and quarrying.” Because IO modeling requires assuming a homogeneous product group, energy flows that are allocated to “mining and quarrying” are distributed to non‐energy supply chains as well. Austria extracts relatively small quantities of natural gas and oil (86 PJ, i.e., 1.8 million metric tons) next to large amounts of non‐metallic (65.5 million metric tons) and to a smaller extent metallic minerals (2.6 million metric tons, the majority of which are iron ores) (UN IRP, 2017). We presume that the monetary flows of “mining and quarrying” products in the official MSUT/MIOT therefore predominantly mirror the flow of non‐energy products. Using energy and non‐energy supply chains alike to allocate energy flows to final demand is a probable cause for disagreements between supply‐extended and use‐extended energy footprints. Relaxing the assumption of homogeneous product groups through further disaggregation of the SRIO model, that is, the underlying MSUT could result in a higher convergence of supply‐extension and use‐extension energy footprints.

Even though MSUT and MIOT are more disaggregated and hence separate monetary flows of energy from non‐energy mining products, like in our GMRIO example EXIOBASE, supply‐extended and use‐extended energy footprints still deviate considerably. This can be interpreted, at least to a certain extent, as an effect of assuming homogeneous energy prices.8In this regard, it is worth mentioning that differences could also be related to misreporting of energy flows by the IEA. It is known that data for non‐OECD countries tend to be of lower quality (IEA, 2019b). In contrast to the supply‐extended IO model, the use‐extended model does not imply any energy price assumptions to allocate energy flows to industries or final consumers that use the energy because all transformations, losses, and distribution processes preceding the use‐phase are implicitly included by design of the use‐extension. It is therefore reasonable to assume that a supply‐extended hybrid IO model where flows of energy products are represented in energy units, meaning no homogeneous prices are assumed for the energy products, could converge more strongly with the results of the use‐extended IO model. Whether supply‐extended and use‐extended energy footprints converge more strongly when using a more disaggregated MIOT or even a mixed‐unit HIOT is beyond the scope of the present paper and should therefore be subject of further empirical investigations.

### 5.5 Two different pre‐analytic perspectives

We agree with Owen et al. (2017) that both extensions are useful and the choice depends on the perspective, (often‐implicit) principles of responsibility and the research question, either focusing on the upstream part (origin/source) or the downstream part (actual energy use of industries) of the energy conversion chain. The observed variations are considerable and emphasize the importance of the extension design when EE‐MIOTs are used for monitoring energy efficiency and short‐ or medium‐term emission reduction targets (Barrett et al., 2013; Barrett, Cooper, Hammond, & Pidgeon, 2018; Scott & Barrett, 2015; Steininger et al., 2014), which usually aim at the same percentage range as the divergences. Which extension is used also reflects an assumption of responsibility in the energy conversion chain and where a demand‐side measure is supposed to intervene in order to curb current unsustainably high levels of energy consumption (Creutzig et al., 2018). If we are concerned with extraction and primary energy supply as prerequisites to any form of energy use and we assume that the extractive sectors are “more responsible,” then we must turn to patterns of energy supply. If, however, we see industrial energy use across sectors as primarily responsible for its own energy use (and emissions), and we view final demand for products as driving energy use of industries, then we may be better advised to consider the distribution of energy use. Accordingly, we would also use different extensions to model the expected impact of measures targeting different stages of the energy conversion chain. Similar discussion can be found in the literature on carbon accounting and (shared) responsibility9The freedom of choice in regard to different extension designs grows, as the general data availability in environmental accounting matures, making it ever more important that researchers are aware of the underlying assumptions taken in the construction of their environmental extension and that they are critically reflected upon and made transparent. Until now, energy IO analysis was predestined to be a pioneer in developing different extension designs, because energy accounts that record the various stages of the conversion chain are commonly reported by national statistical institutes. However, efforts have been undertaken to develop use‐extensions for accounting of biomass flows as well (Ewing et al., 2012). Here it is the material cycle, as the physical counterpart to the energy conversion chain, which is broken down at different stages. Stadler et al. (2018) used substance flow analysis to construct an extension depicting the use of copper products by economic sectors for the Chinese IO table. (Csutora & Vetőné mózner, 2014; Lenzen, Murray, Sack, & Wiedmann, 2007; Steininger, Lininger, Meyer, Muñoz, & Schinko, 2016).

## 6 CONCLUSION

The present study investigated the conceptual and empirical implications of applying two different but frequently used designs of energy extensions. Our empirical comparison of supply‐extended and use‐extended SRIO and GMRIO model results revealed considerable divergences in energy footprints. Analogies can be drawn from lively debates in LCA, which reveal the conceptual differences between the two extension designs and show that although the result of both EE‐MIOTs are usually described using the same terminology, they must be interpreted as providing different types of information. The present work therefore calls for more rigor in communicating and disclosing the design of extensions to allow for more meaningful interpretations of results, greater transparency and better reproducibility of modeling results. Finally, we urge industrial ecologists to critically and transparently reflect on their pre‐analytic view taken on how we (often implicitly) attribute responsibility to derive potentially meaningful interventions into the socio‐economic system. In the end, this also requires us to think about actors, their relations to one another, and their decision‐making power, a crucial aspect for sociometabolic research.

## FUNDING INFORMATION

This project has received funding from the European Research Council (ERC) under the European Union's Horizon 2020 research and innovation programme (grant agreement No. 725525). Anke Schaffartzik acknowledges financial support from the Spanish Ministry of Economy and Competitiveness, through the “María de Maeztu” program for Units of Excellence (MDM‐2015‐0552).

## CONFLICT OF INTEREST

The authors declare no conflict of interest.

## Supporting information


**Supporting Information S1**: This supporting information S1 provides data for figures 2, 3, 4, 5, 6, and 7 and detailed results of the SRIO model of Austria and the MRIO database EXIOBASE in the form of pivots and underlying lists. It also includes SRIO‐ and EXIOBASE codes, a concordance table to match SRIO model and energy accounts (EA).

**Supporting Information S2**: This supporting information S2 includes the following information: Relevant terms and definitions of the SEEA framework, further details on the graph visualization of EE‐MIOTs, a more elaborated description of the SRIO model of Austria and a comparison of the energy footprints for 1999, 2007 and 2014. Furthermore, energy footprints of households and exports are discussed disaggregated by final products and a concordance table between energy products and SRIO industries is shown. The last section gives an overview over the energy extensions and the footprints from the EXIOBASE MRIO
