# Supplementary material for: Supply versus use designs of environmental extensions in input–output analysis: Conceptual and empirical implications for the case of energy
Source: J Ind Ecol. 2019 Dec 17;24(3):548–63. doi: 10.1111/jiec.12975 (PMC7319417; doi:10.1111/jiec.12975)
Supplement: Supplementary file 2 — Supporting Information S2: This supporting information S2 includes the following information: Relevant terms and definitions of the SEEA framework, further details on the graph visualization of EE‐MIOTs, a more elaborated description of the SRIO model of Austria and a comparison of the energy footprints for 1999, 2007 and 2014. Furthermore, energy footprints of households and exports are discussed disaggregated by final products and a concordance table between energy products and SRIO industries is shown. The last section gives an overview over the energy extensions and the footprints from the EXIOBASE MRIO (PDF 1.18 MB) [file 44498_2020_2403008_MOESM2_ESM.pdf]

SUPPORTING INFORMATION FOR:

Wieland, H., S. Giljum, N. Eisenmenger, D. Wiedenhofer, M. Bruckner, A. Schaffartzik, and A. Owen. 2019. Supply versus Use Designs of Environmental Extensions in Input-Output Analysis: Conceptual and Empirical Implications for the Case of Energy. *Journal of Industrial Ecology.*

Summary

This supporting information S2 includes the following information: Relevant terms and definitions of the SEEA framework, further details on the graph visualization of EE-MIOTs, a more elaborated description of the SRIO model of Austria and a comparison of the energy footprints for 1999, 2007 and 2014. Furthermore, energy footprints of households and exports are discussed disaggregated by final products and a concordance table between energy products and SRIO industries is shown. The last section gives an overview over the energy extensions and the footprints from the EXIOBASE MRIO.

1. SEEA: terms and definitions

The System of Environmental and Economic Accounting (SEEA), which was adopted as an international standard by the United Nations Statistical Commission, is a central reference point for the integration of physical and monetary accounts. The framework is based on the definition of three key flows: Flows from the environment into the economy are *natural inputs*, flows within the economy are *products*[1], and flows from the economy to the environment are *residuals*.

Natural inputs encompasses all energy flows that are removed and captured from the environment by economic entities. This includes mineral and energy resources (e.g., crude oil, natural gas, coal, peat, and uranium) and inputs from renewable energy sources (e.g., solar, wind, hydro, geothermal and biomass). *Products* are energy carriers that are produced i.e. generated by an economic entity. This comprises fuels, electricity and heat that is generated and sold to third parties by an economic entity. Energy products include electricity and heat from the combustion of biomass and solid waste. Some energy products may be used for non-energy purposes. For example, naphtha is used in the manufacture of plastic. A distinction must be made between primary and secondary energy products. Primary energy products are produced directly from *natural inputs*; they differ only by the amount of energy lost during production (e.g., natural gas evaporates during extraction). Secondary energy products are the result of a transformation of primary, or other secondary, energy products (e.g., crude oil into petroleum products, or fuel oil into electricity). *Residuals* comprise a number of energy flows. Most focus is on energy losses,

[1] Please note that there are also residual flows within the economy, for example solid, liquid or gaseous wastes that are disposed at controlled landfills. The SEEA framework considers such flows to remain within the economy. For the sake of simplicity, we omit these flows here.

which includes losses through flaring and venting of natural gas and losses during production and transformation processes. Energy losses during distribution may arise from leakages of liquid fuels, loss of heat during transport of steam, and losses during gas distribution, electricity transmission and pipeline transport.

Figure S2-1 displays the energy conversion chain, discerning the following energy flows and processes. Primary energy industries, i.e. producers, capture *natural inputs* from the environment (R) and incinerate solid wastes (W) to produce *primary energy products* (V_P). Part of the input returns back to the environment as *residuals* in the form of transformation losses (L) and primary energy industries' own consumption for heating, pumping, traction, and lighting (O). Secondary energy producers use *primary* or other *secondary energy products* as a transformation input (U_t) to produce *secondary energy products* (V_s). Again, a fraction of the input leaves the process as a *residual* flow and is lost to the environment ($L + O$). Additionally, primary producers' own consumption of *secondary energy products* as well as secondary producers' own consumption of *primary energy products* (U_e) is also accounted on the input side. The total in- and out-flows of the energy industries (transformation processes) are balanced: $R + W + U = L + O + V$.

Figure S2-1a: Simplified visualization of the energy conversion chain where energy flows between industries and markets are depicted as a black box and only boundary flows that enter and leave the system are shown. Figure S2-1b: The energy conversion chain using a graph visualization of an ESUT. To facilitate a better overview, primary and secondary energy production processes are aggregated into one box. Figure adapted from (Pauliuk et al. 2015).

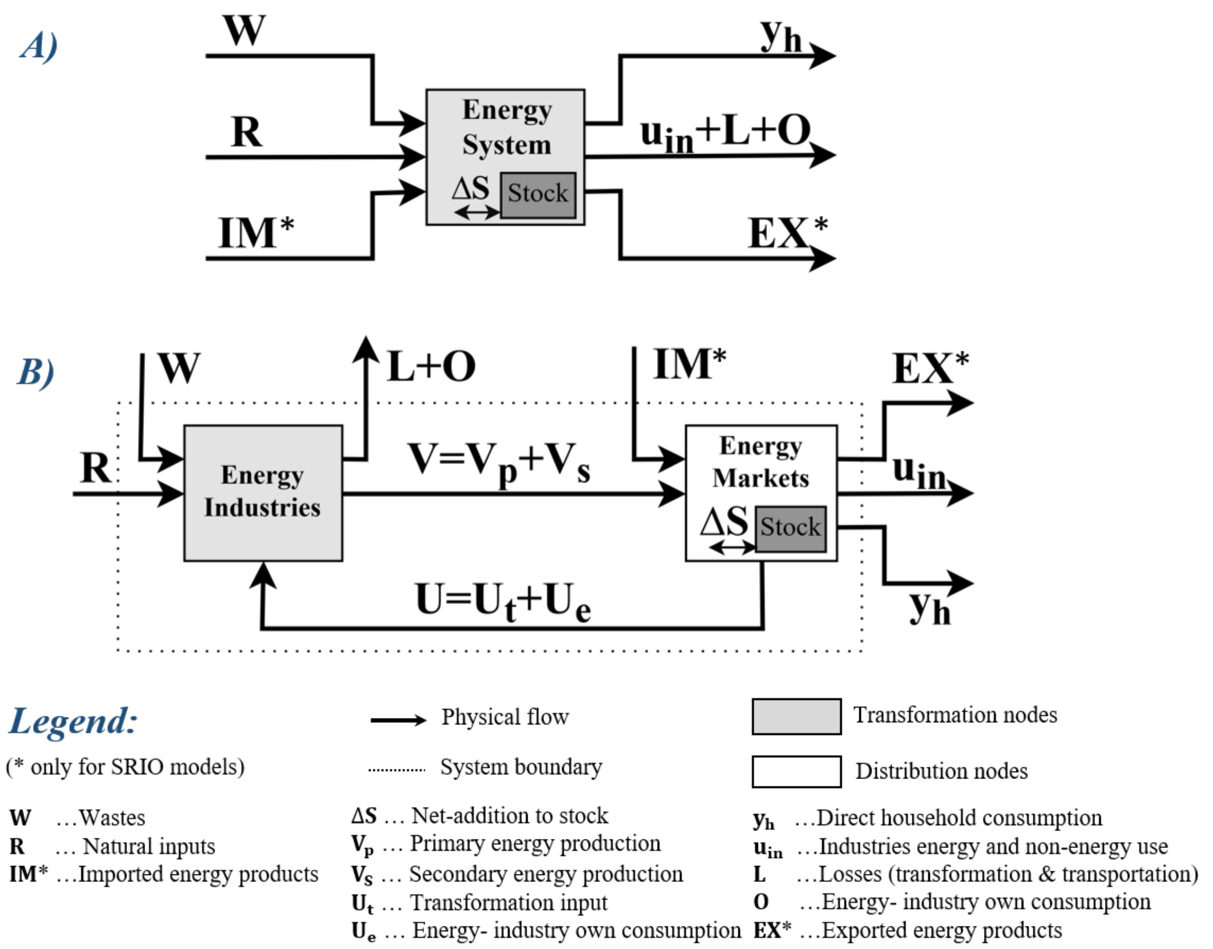

Energy markets for *primary and secondary energy products* receive inputs from domestic ($V = V_p + V_S$) and foreign producers (IM). Markets distribute *energy products* to non-energy industries (u_{in}), households (y_h), domestic energy industries ($U = U_t + U_e$) and foreign industries (EX). Markets add energy to stocks for future use (S_{add}) and withdraw (S_{with}) energy from last year's production respectively ($\Delta S = S_{add} - S_{with}$). Again, the accounting of all inputs to and outputs of the distribution processes (i.e. flows to and from markets) must lead to a balanced system: $V + IM + S_{with} = EX + U + u_{in} + y_h + S_{add}$.

The economic value of energy flows

Physical flows of *natural inputs* and *residuals* are not captured by monetary transactions (UN et al., 2014). All monetary transactions are interactions between economic entities. A monetary transaction is one in which an economic entity makes/receives a payment, incurs a liability or receives an asset stated in monetary units (EC, IMF, OECD, UN, & WB, 2009). Flows from the environment, the dilution of pollutants or the disposal of wastes are considered as "free gifts of nature" (Duchin, 2010; Leontief, 1970). *Natural inputs* and *residuals* are boundary flows and therefore represent an "extension" of the monetary IO table and the scope of the System of National Accounts (SNA). Products (primary and secondary), on the other hand, are flows that result from production processes within the economy. They have an economic value and can thus be measured in both physical and monetary units (UN, EU, FAO, OECD, & WB, 2017).

2. Graph visualisation of EE-MIOTs

Pauliuk and colleagues (2015) illustrate how supply-use tables (SUTs) can be completely described as a bipartite directed graph. Graphs are an established mathematical concept and frequently deployed for describing relations between elements (Diestel, 2017). A graph model consists of edges or arrows that represent flows of objects and vertices (or nodes) that represent the processes in the system. Graphs that have two disjoint sets of nodes and that only have directed edges that connect a node in one set to a node in the other are called bipartite directed graphs. A SUT is a tabular representation of a bipartite directed graph where industries and markets (two disjoint sets of nodes) connect via flows of commodities (directed edges). Industries represent transformation nodes and markets distribution nodes. The former transform input commodities into output commodities, whereas the latter do not transform inputs, but simply transfer the intermediate output of one or more transformation nodes to the transformation nodes that use this intermediate exchange as an input (Pauliuk et al., 2015). In the following, we apply a simplified representation of bipartite directed graphs using boxes (representing nodes) and arrows (flows), a notation that is common in material flows analysis (Bertram et al., 2017; Mao, Dong, & Graedel, 2008).

The next section shows how monetary flows in a single-region MIOT can be described as a bipartite directed graph, which is then followed by an extended graph visualization of the supply-extended and the use-extended SRIO model. The descriptions hereafter supplement the description of the two extension designs from the main text (see materials and method section).

Figure S2-2 presents two variants of the single-region MIOT structure. The upper part shows an aggregated version where energy industries (primary and secondary) and non-energy industries (e.g., manufacturing and services), as well as energy markets and non-energy markets, are lumped together. Vector x stands for the gross production of industries. This is the amount of commodities supplied to the (domestic) market. Gross output x is used either as an intermediate input for production (Z) or for

final consumption (y). y comprises consumption of households, government and non-profit organizations serving households, as well as gross fixed capital formation and changes in inventories and, in a SRIO framework, exports. Industries' payments to primary i.e. factor inputs (or value-added in production) are represented as v. This includes compensation for employees and capital (interest rates), taxes and profits. In the SRIO model of the present study, monetary transactions representing imports (im), either competitive or non-competitive, are endogenized in the MIOT. This means imported commodities are treated as if they were produced within the economy, hence imports are included as part of the gross production vector (x) of the monetary IO table. Industries ($v + im + Z = x$) and markets ($x = Z + y$) are balanced and the expenditures on the consumption side (y) must equal the payments for factors and imports on the production side ($im + v = y$).

The lower part Figure S2-2 shows a second variant of the MIOT where processes and flows are disaggregated into energy industries (subscript e) and non-energy industries (subscript i). The total intermediate use of commodities (Z) is now divided into energy commodities used by energy industries (Z_{ee}), energy commodities used by non-energy industries (Z_{ei}), non-energy commodities used by non-energy industries (Z_{ii}) and non-energy commodities used by energy industries (Z_{ie}). The same division applies to factors of production ($v = v_e + v_i$), commodity imports ($im = im_e + im_i$), gross production of industries ($x = x_e + x_i$) and final consumption ($y = y_e + y_i$).

Figure S2-2: The MIOT structure.

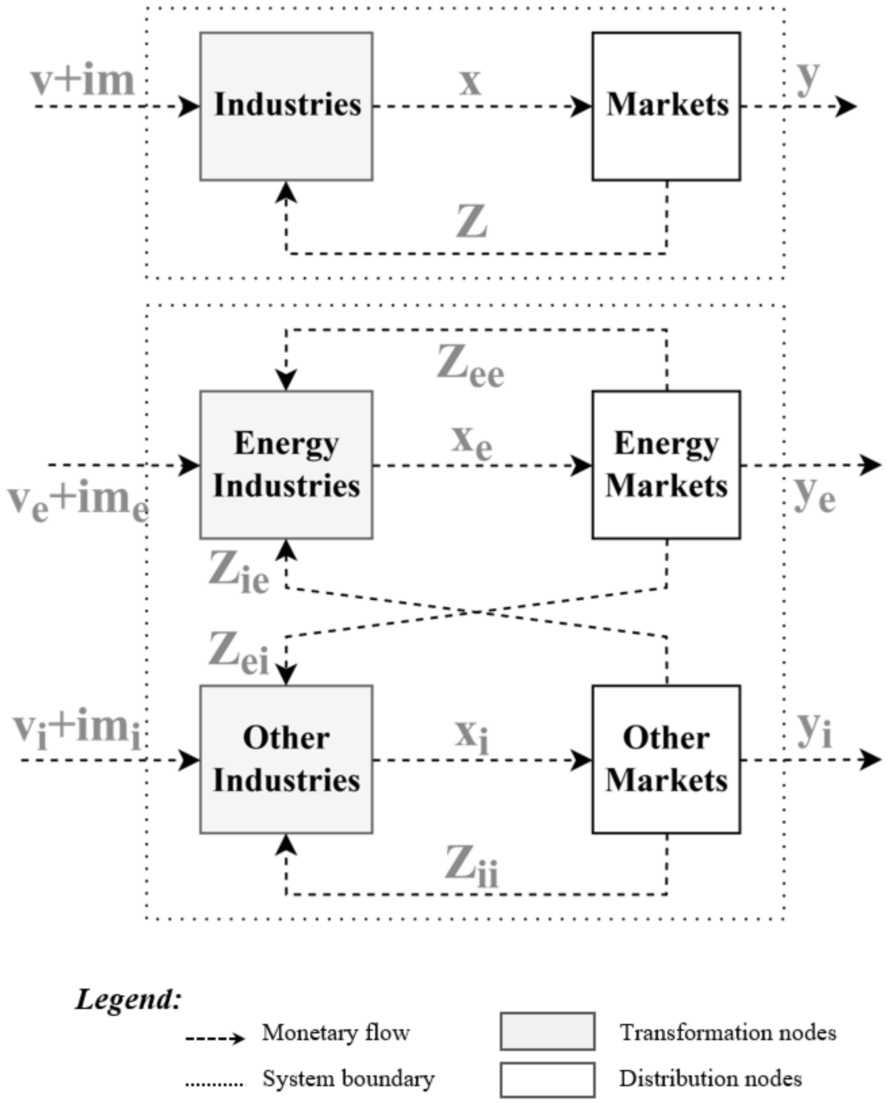

Based on the foregoing, the next figure shows a more disaggregated graph-based representation of the two extension-designs form the perspective of the MIOT. Figure S2-3 distinguishes between IO industries producing energy commodities and industries producing non-energy commodities. Note that here withdrawal (S_with) and addition (S_add) to stocks are explicitly represented as solid arrows in the corresponding extension design. Figure S2-3 depicts the supply-type and Figure S2-3 the use-type extension.

Figure S2-3: The dotted line visualizes the system boundary of the single-region MIOT. Dashed arrows stand for monetary transactions and solid arrows for energy flows. Grey boxes represent transformation nodes, white boxes are distribution nodes (markets) and the three boxes on the right stand for the three final demand categories for which the energy accounts report consumption.

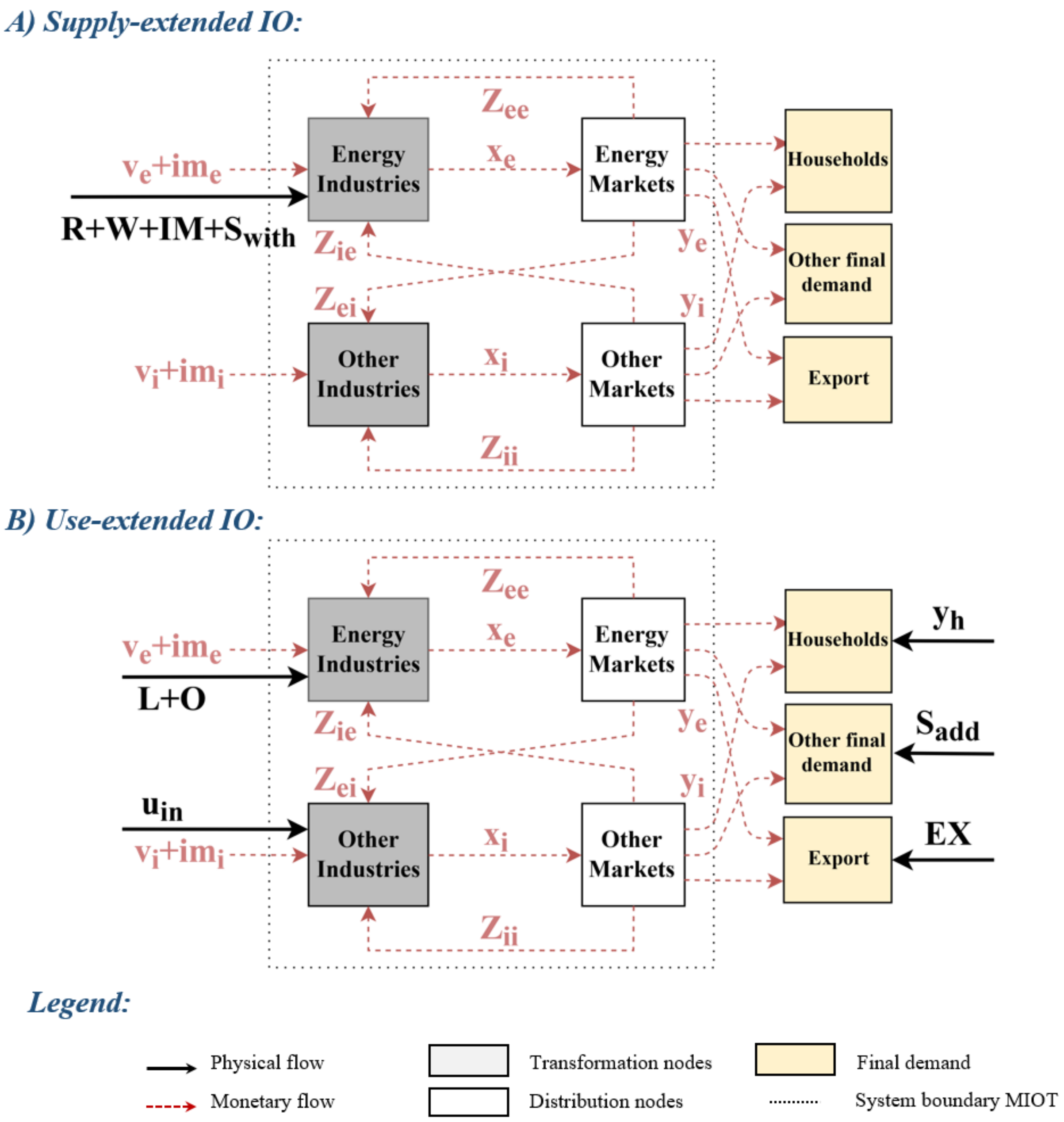

3. Detailed description of the single-region IO model of Austria

Monetary Supply-Use tables (mSUTs) form the basis of the single-region IO model of Austria. The construction of the IO model follows the general description of the commodity-by-industry-approach as

set forth in chapter five in Miller and Blair (2009). The operational form of a model that applies an industry technology assumption is $x = [D(I - BD)^{-1}]Yi$, where I is the identity matrix, x the vector of total industry output, Y the matrix of commodity final demand, i an summation vector containing ones, D the matrix of *commodity output proportions* and B the *commodity-by-industry coefficient* matrix. BD is the commodity-by-industry-approach counterpart to the *technology matrix* (A) of the original input-output model. It follows that $(I - BD)^{-1}$ is a *commodity-by-commodity total requirement* matrix and thus equivalent to the Leontief Inverse L. Pre-multiplication with D, which can be seen as a "concordance" or bridge table between total commodity output (q) and total industry output ($x = Dq$), results in an *industry-by-commodity total requirement* matrix $[D(I - BD)^{-1}]$ which connects commodity final demand (y) to total industry output (x). The following section describes the mSUTs and how they were applied to construct the above-mentioned matrices.

The mSUTs for Austria differentiate between 65 commodities and 65 industries (Statistics Austria 2014). The Supply table (S), with the dimensions *commodity by industry*, is valued in basic i.e. producer prices. Element $s_{i,j}$ shows the value of the output of commodity i that is produced by the domestic industry j. Three column vectors that account for trade and transport margins (m), added taxes less subsidies (t) and imports (im) extend the domestic Supply table. Summing over the rows of the extended Supply table yields the total supply of commodities (q) in purchasers' prices: $q = S\,i + m + t + im$, where i is an appropriate summation vector containing ones. Dividing the rows of S by the corresponding row sum and transposing the result yields the *commodity output proportions* matrix $D = \left(\widehat{Si}^{-1}\,S\right)'$, where i is an summation vector containing ones, ^ indicates the diagonalization of a vector, $^{-1}$ a matrix inversion and superscript ' the transposition of a matrix. Element d_{ij} shows the relative share of domestic production of commodity j that is produced by industry i. Subsequently, we can calculate the total industry output in purchasers' prices via $x = Dq$. Please note that x comprises domestic industry production (S) and imported commodities (im), assuming that imported commodities have the same industry source as commodities from domestic production.

The Use table (U), with the dimensions *commodity by industry*, is valued in purchasers' prices and does not differentiate between commodities from domestic (S) and foreign production (im)[2]. Element u_{ij} shows the value of commodity i that is utilized as an intermediate input by industry j. Dividing the Use table column-wise by the total industry output (i.e. supply) results in the *commodity-by-industry coefficient* matrix $B = U\hat{x}^{-1}$. Element b_{ij} shows the amount (or value) of intermediate inputs of commodity i that is directly used by industry j to produce a unit of its output. Post-multiplication with D yields a *commodity-by-commodity coefficient* matrix ($A_{com} = BD$) and pre-multiplication an *industry-by-industry coefficient* matrix ($A_{ind} = DB$). Additionally, transaction/IO matrices can be derived from the *coefficient* matrices through multiplication with output vectors. For *commodity-by-commodity*

[2] This is what Majeau-Bettez and colleagues call an *untraceable*, because two-dimensional, Use table ((2014))

$Z_{com} = DB\hat{q}$ and for *industry-by-industry* this is $Z_{ind} = DB\hat{x}$. The following figure S2-4 shows the industry-by-industry variant of the transaction matrix, i.e. IO table, of the model: $Z_{ind} = DB\hat{x}$.

Figure S2-4a: Aggregated map of the industry-by-industry IO table of Austria for the year 2014 according to the MIOT structure. All flows that cross or fall within the system boundary are part of and thus depicted by the MIOT: v stands for value added (GDP), im for imports, Z for intermediate use, x for deliveries to markets and y for final use. Figure S2-4b: A disaggregated map of the industry-by-industry IO table of Austria for the year 2014 according to the MIOT structure distinguishing between industries producing energy commodities and industries producing non-energy commodities. UNIT: Billion Euro. Like Sankey flows, the size of the arrows represent the monetary value.

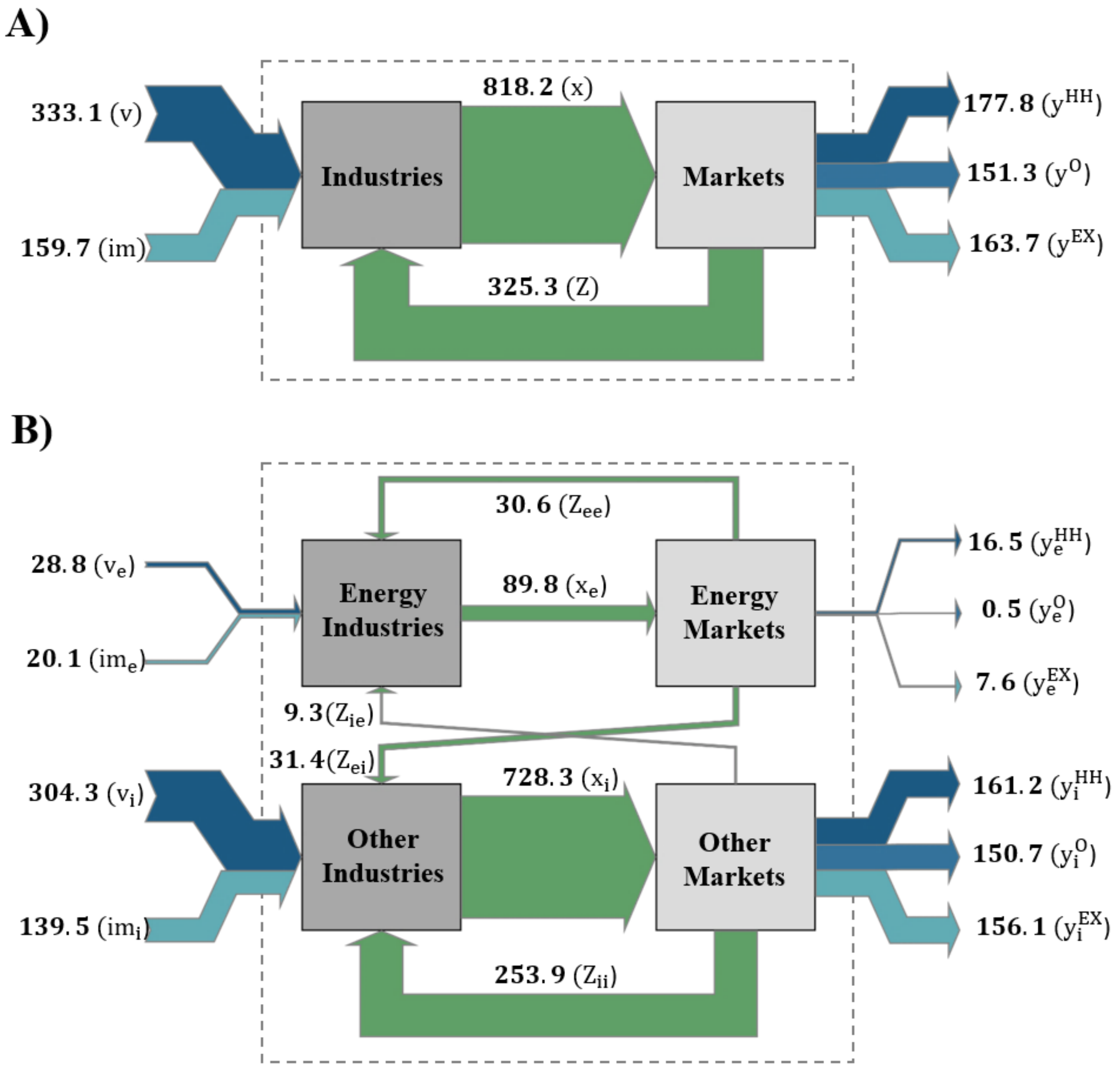

4. Energy footprints for 1999, 2007 and 2014

Comparing the final demand footprints and the associated variations for the years 1999, 2007 and 2014 reveals a relatively stable pattern over time. Figure S2-5 shows that the footprints of households and *other final demand* were always larger when applying a supply-extended IO model and the ones of

export demand consequently always smaller. In other words, the energy footprints of export demand were always larger when applying a use-extended IO model. Across all years, the strongest convergence between the two model results, viewed in both relative and absolute terms, is found for the footprints of households. The household footprints of 2007 were most similar showing an absolute difference of only 48 PJ or 7%, when taking the hypothetical mean of the two results as a benchmark. In absolute terms, again export footprints were most dissimilar. However, the observation that the ranking of the final demand footprints is sensitive to the extension design, does not apply to the results of 1999. For this year, both IO models rank the categories in the same order.

Figure S2-5: Energy footprints by final demand categories calculated with the SUP-extended (solid line) and the USE-extended (dashed line) IO model for 1999, 2007 and 2014. The household footprints (diamonds) at the top, the footprint of other domestic final demand (triangles) at the bottom and the footprints of export demand (circles) in-between.

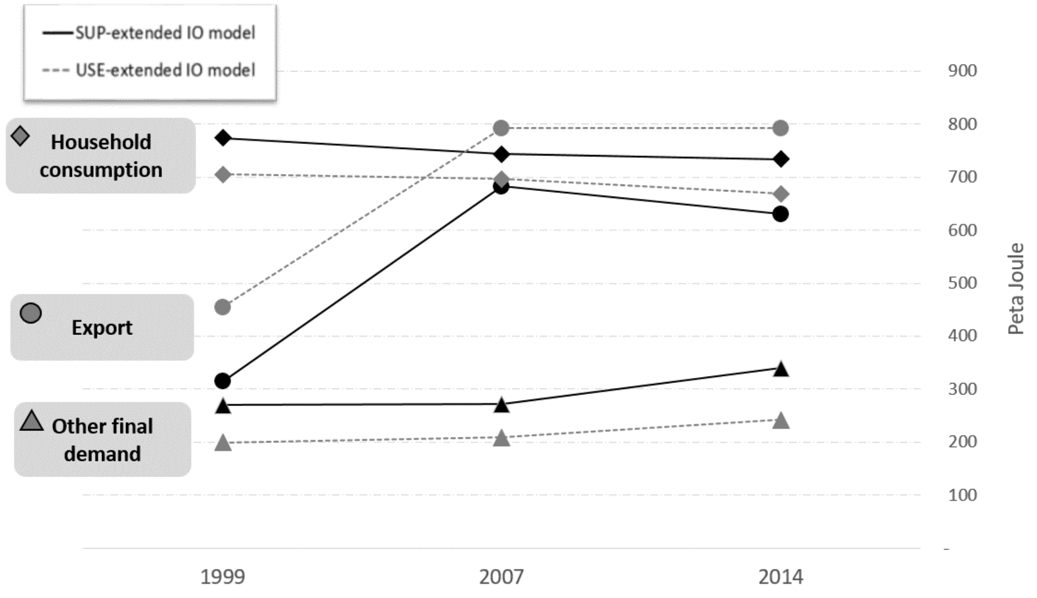

5. Energy footprints of households and exports disaggregated by final products

This section gives a brief overview of the differences between the energy footprints of household (Figure S2-6) and export demand (Figure S2-7) disaggregated by products. The waterfall-charts shows the top-12 product footprint differences sorted in descending order from left to right, where the products with the largest absolute/gross difference, for example for the household footprint this is *real estate services* (68) with a difference of 23 PJ, is on the far left. Please note that all products not explicitly presented in this figure (hence aggregated in NEC) have an absolute difference that is below the smallest product difference shown here. The waterfall chart reveals how the differences on the product level accumulate and cancel each other out in the aggregated household footprint. The dark bars indicate that the supply-

extended product footprint is larger than the use-extended product footprint whereas a lighter hatched bar stands for the opposite. The arrow stands for the total difference between the footprints.

Figure S2-6: Comparing the energy footprints of households for the 2014 broken down by final products. The left side shows the total energy footprints as calculated with the SUP-extended and the USE-extended IO model. The right side shows a waterfall chart with top-12.

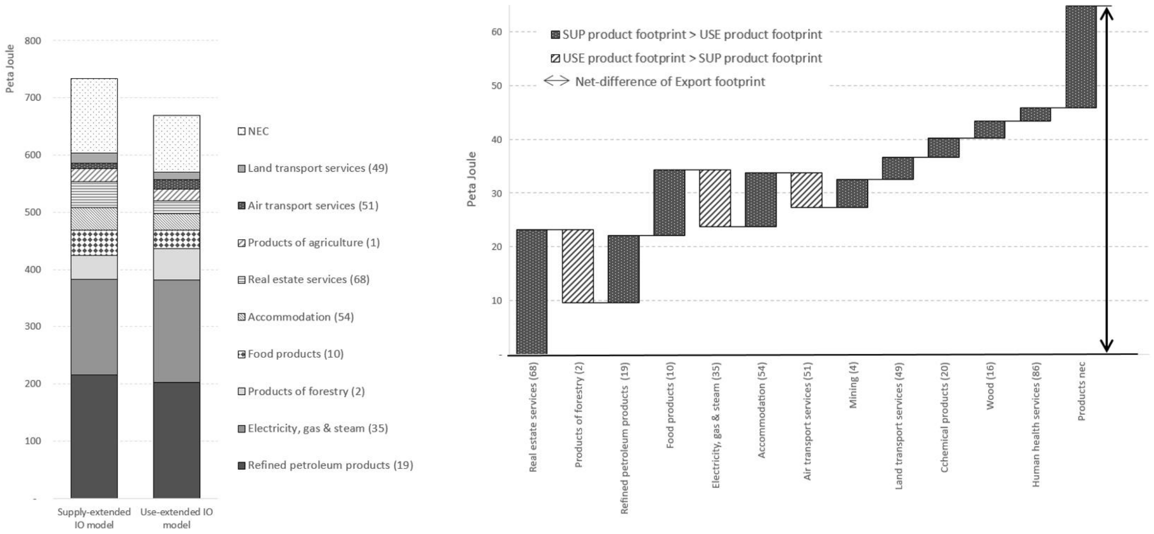

Figure S2-7: Comparing the energy footprints of exports for the 2014 broken down by final products. The left side shows the total energy footprints as calculated with the SUP-extended and the USE-extended IO model. The right side shows a waterfall chart with top-12 product footprint differences sorted in descending order.

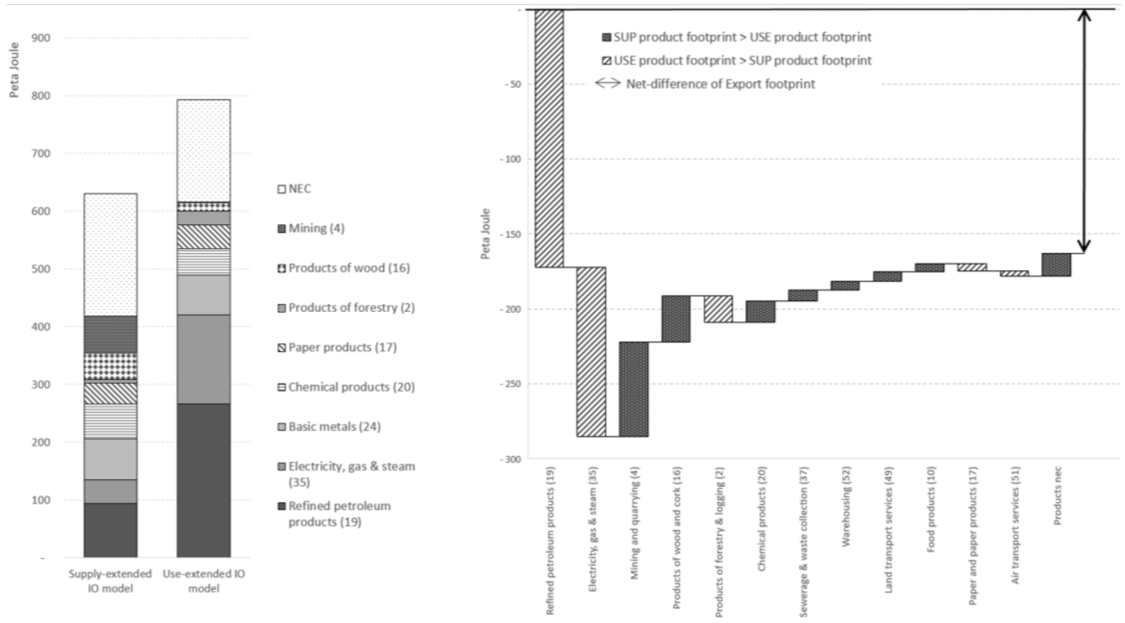

Disaggregating the footprints by final products reveals that only a small number of products makes up the largest part. In the case of households, for both model results, the same six products account for at least 75% of the total energy footprint. This is refined petroleum products with approx. 215 PJ in the supply-extended model and 203 PJ in the use-extended model, electricity with 168 PJ and 178 PJ respectively, products of forestry with 42 PJ and 55 PJ, food products with 44 PJ and 31 PJ, accommodation services with 40 PJ and 30 PJ and real estate services with 45 PJ and 22 PJ (see SI.2-5 and the result sheet in SI.1 for more details). In the case of exports, which is the final demand category with the largest absolute footprint deviation, the same final products stand out but with more pronounced absolute differences. For example, in both model results, refined petroleum products show the largest energy footprint, yet the supply-extended model allocates only approx. 95 PJ whereas the use-extended model allocates 267 PJ, a difference of 172 PJ. In both model calculations, this is followed by electricity with 41 PJ and 154 PJ respectively. The differences in these two products are mainly due to the vast amount of direct energy exports of fuels (248 PJ) and electricity (146 PJ) allocated in the use-extended model. However, large differences on the product level partly offset each other when summed up to the footprints of final demand categories.

6. Concordance table between energy products and IO industries.

Because official Energy Accounts do not provide an allocation of energy supply, energy loss and non-energy use to IO industries, the following adjustments were made. Firstly, a concordance table was constructed (Table S2-1) that links the supply of energy products to IO i.e. NACE industries using the statistical classification of products by activity (CPA) (Statistics Austria, 2004). Secondly, according to the information contained in the more detailed raw data set, only four manufacturing industries (producing petrochemicals, rubber, glass and basic metal products) are using energy products for non-energy purposes. Lacking detailed information, total non-energy use was therefore allocated to these four industries assuming a constant ratio between final energy consumption and non-energy use. Thirdly, transformation and transportation losses were allocated to the IO industries producing the energy products.

Table S2-1: Concordance table between energy products and NACE industries of the IO model.

Name	Code	Energy products
Agriculture	1	Biofuels
Forestry	2	Fuelwood
Mining of coal and lignite	10	Hard coal, Brown coal<br>BKB, Fuel peat
Extraction of crude petroleum and natural gas	11	Crude oil, Natural gas
Manufacture of coke, refined petroleum products	23	Coke, Refinery feedstock nec, Fuel, Kerosene, Diesel , gasoil for heating, fuel oil, liquid gas, petrochemical products nec, Refinery gas , Coke oven gas
Manufacture of basic metals	27	Blast furnance gas
Recycling	37	Combustible waste
Electricity, gas, steam and hot water supply	40	Geothermal, District heating, Electric energy<br>Hydropower, Wind and PV

7. Comparison of energy footprints calculated with EXIOBASE

EXIOBASE is a global multi-region input-output database that includes 44 countries and 5 aggregated rest of the world regions (see country codes in SI.1) for the years 1995 to 2016. The industry-by-industry table differentiates between 163 sectors (Tukker, Giljum, & Wood, 2018; Wood et al., 2015). The environmental extension includes various energy flows, for example losses or direct consumption of households, for 74 energy carriers.

Figure S2-8: Overview of energy flows included in the extensions of the EXIOBASE database.

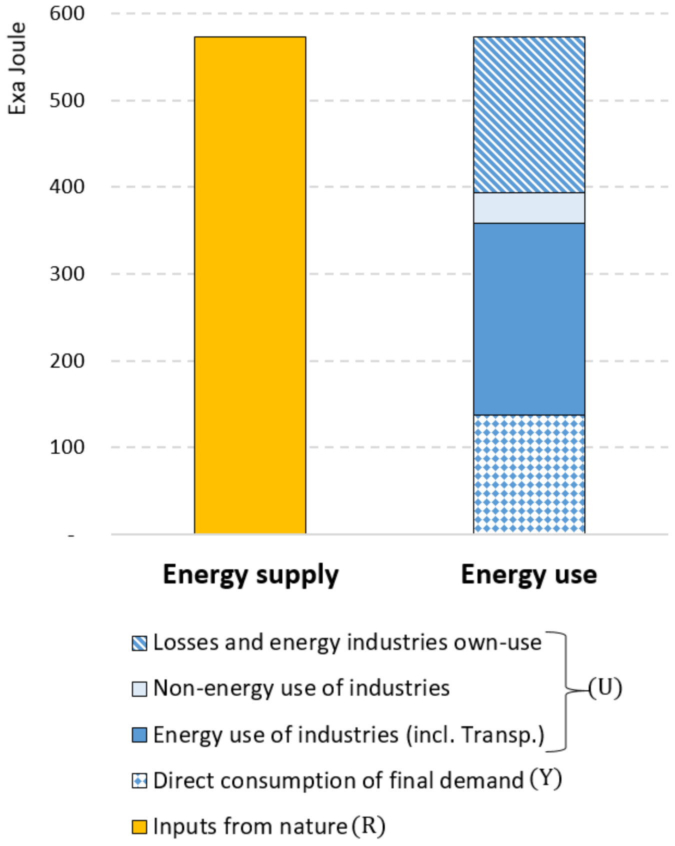

As can be seen in Figure S2-8, according to the energy supply-use extensions of EXIOBASE, in 2014 total natural inputs (R) into the global economy amounted to 573.4 EJ (Exa Joule). Global energy use of industries (U) - which includes transformation and transportation losses, energy-industries own use and non-energy use - was 434.8 EJ (75.1% of global natural inputs). Direct energy consumption for final use (Y) - which comprises demand of households, governments, non-profit organisations and changes in inventories – was 137 EJ (24.9% of global natural inputs). Losses and energy-industries own use amounted to 179.9 EJ and non-energy use to 34.6 EJ in 2014.

Table S2-2 shows the energy footprints of regions as calculated with EXIOBASE. Summation of all supply-extended and all use-extended energy footprints must add up to the same total of 573.4 EJ.

Table S2-2: Overview of supply-extended and use-extended energy footprints of nations, 2014, EXIOBASE, UNIT: Peta Joule.

Country	Supply-extended footprint	Use-extended footprint	Absolute difference	% of SUP-footprint	% of USE-footprint	Within 15% threshold?
**Austria**	1 339	1722	383	29%	22%	NO
**Belgium**	2 190	2 426	236	11%	10%	YES
**Bulgaria**	820	746	74	9%	10%	YES
**Cyprus**	102	177	75	74%	43%	NO
**Czech Republic**	1 574	1 688	114	7%	7%	YES
**Germany**	13 604	16 545	2 941	22%	18%	NO
**Denmark**	957	1 105	148	16%	13%	NO
**Estonia**	365	256	109	30%	42%	NO
**Spain**	4 626	5 261	635	14%	12%	YES
**Finland**	1 262	1 677	415	33%	25%	NO
**France**	8 124	12 582	4 458	55%	35%	NO
**Greece**	2 033	1 845	188	9%	10%	YES
**Croatia**	334	344	10	3%	3%	YES
**Hungary**	2 032	1 383	649	32%	47%	NO
**Ireland**	917	884	32	4%	4%	YES
**Italy**	10 379	8 010	2 368	23%	30%	NO
**Lithuania**	774	419	355	46%	85%	NO
**Luxembourg**	286	230	56	20%	25%	NO
**Latvia**	240	243	4	2%	2%	YES
**Malta**	134	87	47	35%	53%	NO
**Netherlands**	3 258	4 143	885	27%	21%	NO
**Poland**	3 939	4 328	389	10%	9%	YES
**Portugal**	913	966	53	6%	5%	YES
**Romania**	1 285	1 448	163	13%	11%	YES
**Sweden**	1 690	2 245	555	33%	25%	NO
**Slovenia**	309	309	0	0%	0%	YES
**Slovakia**	930	882	48	5%	5%	YES
**United Kingdom**	10 256	11 657	1 400	14%	12%	YES

Country	Supply-extended footprint	Use-extended footprint	Absolute difference	% of SUP-footprint	% of USE-footprint	Within 15% threshold?
**United States**	92 751	104 237	11 486	12%	11%	YES
**Japan**	16 554	20 961	4 407	27%	21%	NO
**China**	122 636	113 608	9 028	7%	8%	YES
**Canada**	18 600	11 530	7 070	38%	61%	NO
**South Korea**	8 516	9 667	1 151	14%	12%	YES
**Brazil**	14 476	13 126	1 350	9%	10%	YES
**India**	29 566	35 673	6 107	21%	17%	NO
**Mexico**	9 528	8 469	1 059	11%	13%	YES
**Russia**	20 398	23 147	2 749	13%	12%	YES
**Australia**	11 981	5 319	6 662	56%	125%	NO
**Switzerland**	1 478	1 765	286	19%	16%	NO
**Turkey**	4 899	6 281	1 382	28%	22%	NO
**Taiwan**	5 125	4 110	1 016	20%	25%	NO
**Norway**	2 888	1 478	1 410	49%	95%	NO
**Indonesia**	10 893	8 828	2 065	19%	23%	NO
**South Africa**	4 165	4 343	178	4%	4%	YES
**RoW Asia & Pacific**	46 129	36 249	9 880	21%	27%	NO
**RoW America**	18 826	17 844	981	5%	6%	YES
**RoW Europe**	6 567	6 641	74	1%	1%	YES
**RoW Africa**	25 825	24 337	1 488	6%	6%	YES
**RoW Middle East**	26 997	30 675	3 679	14%	12%	YES

References

Bertram, M., Ramkumar, S., Rechberger, H., Rombach, G., Bayliss, C., Martchek, K. J., . . . Liu, G. (2017). A regionally-linked, dynamic material flow modelling tool for rolled, extruded and cast aluminium products. *Resources, Conservation and Recycling*, *125*, 48–69. https://doi.org/10.1016/j.resconrec.2017.05.014

Diestel, R. (2017). *Graph Theory* (Fifth Edition). *Graduate Texts in Mathematics: Vol. 173*. Berlin, Heidelberg: Springer. Retrieved from http://dx.doi.org/10.1007/978-3-662-53622-3 https://doi.org/10.1007/978-3-662-53622-3

Duchin, F. (2010). Input-Output Economics and Material Flows. In S. Suh (Ed.), *Eco-Efficiency in Industry and Science: v. 23. Handbook on input-output economics for industrial ecology* (Vol. 23, pp. 23–41). Dordrecht, London: Springer. https://doi.org/10.1007/978-1-4020-5737-3_2

EC, IMF, OECD, UN, & WB. (2009). *System of National Accounts*. New York. Retrieved from European Commission; International Monetary Fund; Organisation for Economic Co-operation and Development; United Nations; World Bank website: https://unstats.un.org/unsd/nationalaccount/docs/sna2008.pdf

Leontief, W. (1970). Environmental Repercussions and the Economic Structure: An Input-Output Approach. *The Review of Economics and Statistics*, 262–271.

Majeau-Bettez, G., Wood, R., & Strømman, A. H. (2014). Unified Theory of Allocations and Constructs in Life Cycle Assessment and Input-Output Analysis. *Journal of Industrial Ecology*, *18*(5), 747–770. https://doi.org/10.1111/jiec.12142

Mao, J. S., Dong, J., & Graedel, T. E. (2008). The multilevel cycle of anthropogenic lead. *Resources, Conservation and Recycling*, *52*(8-9), 1050–1057. https://doi.org/10.1016/j.resconrec.2008.04.005

Pauliuk, S., Majeau-Bettez, G., & Müller, D. B. (2015). A General System Structure and Accounting Framework for Socioeconomic Metabolism. *Journal of Industrial Ecology*, *19*(5), 728–741. https://doi.org/10.1111/jiec.12306

Statistics Austria. (2004). *GRUNDSYSTEMATIK DER GÜTER ÖCPA 2002: Einführung Grundstruktur, Erläuterungen*. Vienna.

Tukker, A., Giljum, S., & Wood, R. (2018). Recent Progress in Assessment of Resource Efficiency and Environmental Impacts Embodied in Trade: An Introduction to this Special Issue. *Journal of Industrial Ecology*, *22*(3), 489–501. https://doi.org/10.1111/jiec.12736

UN, EU, FAO, IMF, OECD, & WB. (2014). *System of Environmental-Economic Accounting 2012: Central framework*. New York, Luxembourg. Retrieved from United Nations; European Union; Food and Agriculture Organization of the United Nations; International Monetary Fund; Organisation for Economic Co-operation and Development; World Bank website: https://seea.un.org/content/seea-central-framework

UN, EU, FAO, OECD, & WB. (2017). *System of Environmental-Economic Accounting 2012: Applications and Extensions*. New York.

Wood, R., Stadler, K., Bulavskaya, T., Lutter, S., Giljum, S., Koning, A. de, . . . Tukker, A. (2015). Global Sustainability Accounting—Developing EXIOBASE for Multi-Regional Footprint Analysis. *Sustainability*, *7*(1), 138–163. https://doi.org/10.3390/su7010138
